# Ambient and Dosed Exposure to Quaternary Ammonium Disinfectants Causes Neural Tube Defects in Rodents

**DOI:** 10.1002/bdr2.1064

**Published:** 2017-06-15

**Authors:** Terry C. Hrubec, Vanessa E. Melin, Caroline S. Shea, Elizabeth E. Ferguson, Craig Garofola, Claire M. Repine, Tyler W. Chapman, Hiral R. Patel, Reza M. Razvi, Jesse E. Sugrue, Haritha Potineni, Geraldine Magnin-Bissel, Patricia A. Hunt

**Affiliations:** 1 E. Via College of Osteopathic Medicine - Virginia Campus, Blacksburg, Virginia; 2 Department of Biomedical Sciences and Pathobiology, VA-MD College of Veterinary Medicine, Virginia Tech, Blacksburg, Virginia; 3 School of Molecular Biosciences, Washington State University, Pullman, Washington

**Keywords:** neural tube defects, QACs, QAC disinfectants, teratogenesis, environmental contaminants, abnormal development

## Abstract

**Background:**

Quaternary ammonium compounds are a large class of chemicals used for their antimicrobial and antistatic properties. Two common quaternary ammonium compounds, alkyldimethylbenzyl ammonium chloride (ADBAC) and didecyldimethyl ammonium chloride (DDAC), are combined in common cleaners and disinfectants. Introduction of a cleaner containing ADBAC+DDAC in the vivarium caused neural tube defects (NTDs) in mice and rats.

**Methods:**

To further evaluate this finding, male and female mice were dosed in the feed at 60 or 120 mg/kg/day, or by oral gavage at 7.5, 15, or 30 mg/kg ADBAC+DDAC. Mice also received ambient exposure to ADBAC+DDAC from the disinfectant used in the mouse room. Embryos were evaluated on gestational day 10 for NTDs, and fetuses were evaluated on gestational day 18 for gross and skeletal malformations.

**Results:**

We found increased NTDs with exposure to ADBAC+DDAC in both rats and mice. The NTDs persisted for two generations after cessation of exposure. Notably, male exposure alone was sufficient to cause NTDs. Equally significant, ambient exposure from disinfectant use in the vivarium, influenced the levels of NTDs to a greater extent than oral dosing. No gross or significant axial skeletal malformations were observed in late gestation fetuses. Placental abnormalities and late gestation fetal deaths were increased at 120 mg/kg/day, which might explain the lack of malformations observed in late gestation fetuses.

**Conclusion:**

These results demonstrate that ADBAC+DDAC in combination are teratogenic to rodents. Given the increased use of these disinfectants, further evaluation of their safety in humans and their contribution to health and disease is essential.

## Introduction

Quaternary ammonium compounds (QACs) are a large class of chemicals used for their antimicrobial and antistatic properties. They are common ingredients in cleaners and disinfectants, hand wipes, food preservatives, swimming pool treatments, laundry products, shampoos, conditioners, eye drops, and other personal care products. QACs have been in use for over 60 years, but the number of products containing QACs has increased recently as the versatility of these compounds is recognized. Over time, the chemical structure has been altered to increase antimicrobial and surfactant efficacy resulting in multiple generations of QACs. Many products now contain a combination of two or more QACs. In general, QACs have been considered relatively safe. Published studies on the toxicity of single QACs are limited, and there are no studies examining the teratogenicity of QAC combinations. Because chemical mixtures can act synergistically to produce greater toxic effects than the sum of the individual components, evaluation of common mixtures is essential in the evaluation of chemical risk (Hermens et al., 1985; Faust et al., 2003; Narotsky et al., 2011).

Our interest with QACs began when both the Hunt and Hrubec Laboratories independently noted abrupt declines in mouse colony productivity, along with declines in fetal health, that coincided with the introduction of disinfectants containing the QACs, alkyl (60% C14, 25% C12, 15% C16) dimethyl benzyl ammonium chloride (ADBAC) and didecyl dimethyl ammonium chloride (DDAC). The first instance occurred after relocation of the Hunt laboratory to Washington State University (WSU) with the onset of poor pregnancy rates, late fetal demise, and increased dystocia. The WSU experience provided important preliminary data that were briefly discussed in a published interview (Hunt, 2008). Several years later, the Hrubec laboratory at Virginia Tech (VA Tech) encountered breeding problems and neural tube birth defects (NTDs) that began shortly after a change in room disinfectants. The experiences in both the Hunt and Hrubec labs pointed to the QAC disinfectant but could not confirm toxicity because neither incident tested QACs under experimental conditions.

The literature contained no published studies evaluating developmental or reproductive toxicity of ADBAC+DDAC. To fill this data gap, we evaluated the reproductive and developmental toxicity of these common disinfectants. Reproductive studies demonstrated that QACs adversely affect both male and female fertility and fecundity in rodents (Melin et al., 2014, 2016). In this study, we present our findings on the developmental toxicity of a common formulation mixture of ABDAC+DDAC found in many household and industrial cleaning products.

## Materials and Methods

### FACILITIES

**FIGURE 1:**
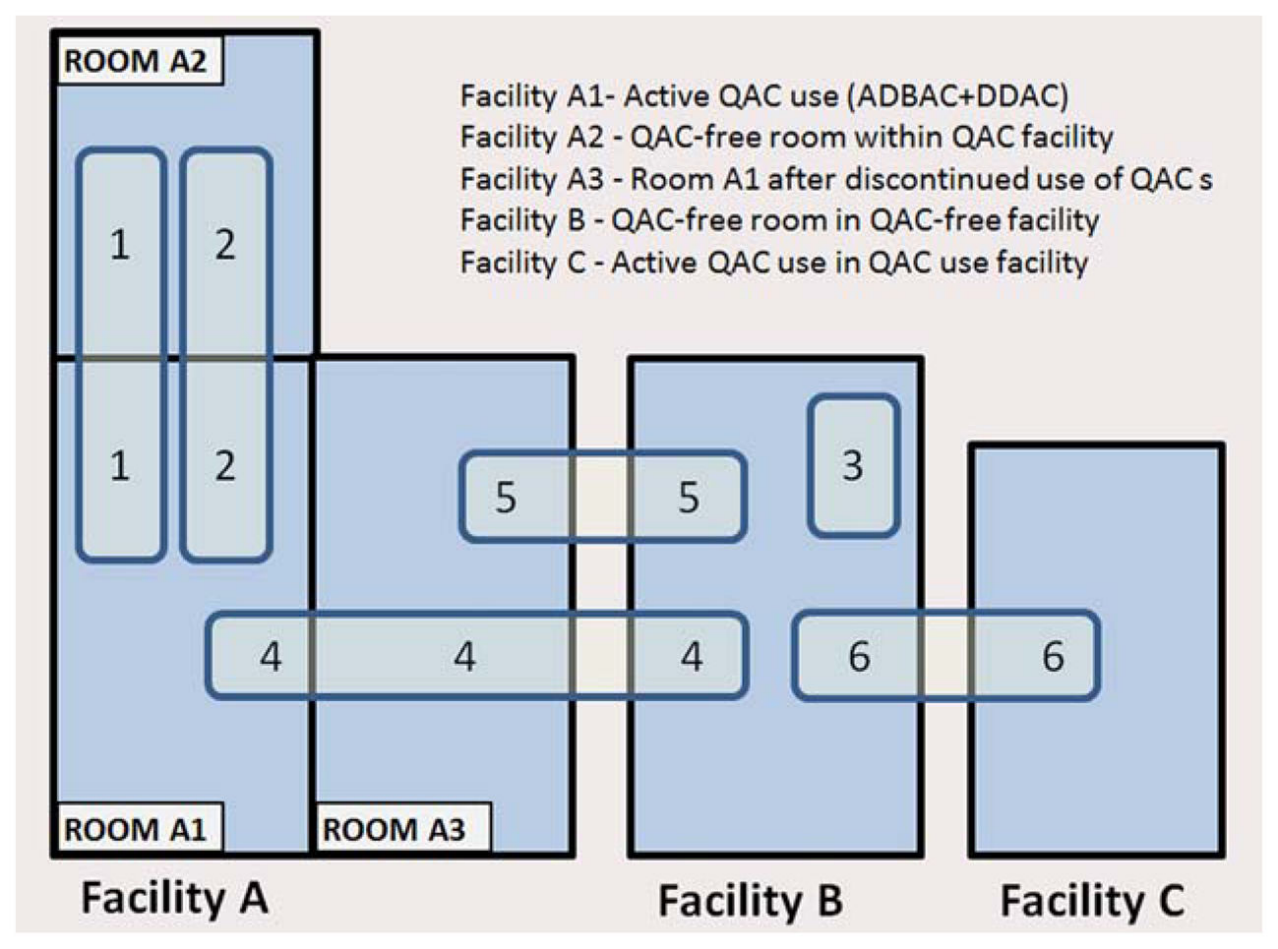
Diagram depicting the animal facilities and rooms used in experiments. In all, six experiments were conducted, numbered 1 to 6. Three animal facilities were used, indicated as Facility A, B, or C. Within Facility A, three rooms were used, indicated as Room A1, A2, or A3. The numbers within the light blue rectangles represents the experiment number. The location of the numbers corresponds to the housing location of a treatment group. For example, in Experiment 5, control mice were held in Facility B and exposed mice in Facility A room 3.

Because ambient exposure from disinfectant use in the mouse room had a greater impact on NTD outcome than the dosing regimens, the facilities and QAC usage therein are described in detail as follows. Five rooms in three separate animal facilities were used during the course of the study (Table 1; Fig. 1). NTDs were first observed (experiment 1, abbreviated E1) in control mice and rats housed in Facility A-Room 1 (abbreviated Room A1), which used ADBAC+D-DAC disinfectant. To prevent ambient exposure, control animals were moved to an adjacent room (Facility A-Room 2, abbreviated Room A2), which was thoroughly cleaned to remove QAC residues and ethanol was used as the disinfectant. Thus, for experiment 2 (abbreviated E2), control and exposed animals were housed in Room A2 and Room A1, respectively; however, when analysis of caging materials indicated contamination in Room A2, mice were moved to a separate facility (Facility B) where only chlorine dioxide disinfectant was used. Breeding stock and control mice for all subsequent experiments were maintained in Facility B. Exposed mice for E4 were housed in Room A1; however, midway through E4 the use of the QAC disinfectant in Room A1 was discontinued in the facility, to denote this, the room was redesignated as Room A3. Because the cessation of QAC disinfectant use in the mouse room significantly reduced the incidence of NTDs in exposed mice in E5, exposures for E6 were conducted in a third facility (Facility C) where ADBAC+DDAC disinfectants remained in use.

In all animal rooms, personnel donned hair bonnets, face masks, disposable gowns, gloves, and dedicated footwear. Additionally, to reduce potential ADBAC+DDAC contamination, control rooms were entered first and not reentered the same day. All rooms were climate-controlled with a 12-hr light/dark cycle, 20 to 25 °C, and 30 to 60% relative humidity. All rooms were serviced by the same animal care staff following the same husbandry procedures in each building.

### ANIMALS

All animal experiments were approved by the Institutional Animal Care and Use Committee at the VA-MD College of Veterinary Medicine, VA Tech, an AAALAC accredited facility. The initial CD-1 mice and Sprague Dawley rats were purchased (Charles River Labs, Raleigh, NC). After initial observation of NTDs in rats and mice, new animals were purchased and were raised for 2 generations. QAC-free breeding stock were re-derived once or twice each year to prevent inbreeding and genetic drift. For all experiments, mice were housed in disposable caging (Innovive, San Diego, CA) and provided distilled water. Rats were housed in new polycarbonate caging. Mice and rats were fed standard rodent diet (Teklad 7013, Envigo, Indianapolis, IN). Exposed and control mice for E2 and E4 were fed a gel diet (Bio-Serv, Frenchtown, NJ) prepared according to manufacturer instructions. For breeding, mice were paired two or three females to one unrelated male while rats were paired one to one. After pairing for breeding, females were checked for copulation plugs each morning. When a sperm plug was found, dams were designated as gestational day (GD) 0.

### QAC DOSING

For E2 and E4, mice were dosed by adding 60 or 120 mg ADBAC+DDAC/kg body weight/day of commercial disinfectant (Labsan 256-cpq, Sanitation Strategies, Holt, MI) in Nutra-gel diet. Doses below the LOAEL were selected as described in Melin et al. (2014). Doses were calculated based on the sum of active ingredient in the disinfectant (6.76% ADBAC [60% C-14, 25% C-12, 15% C-16] and 10.1% DDAC), with an average daily food consumption of 28% body weight as described in Melin et al. (2014). An excess of gel food was provided fresh daily so as not to restrict food intake, and intake was monitored daily. There was no difference in food consumption between treatment groups (data not shown).

Mice were acclimated to undosed gel diet for 1 week before dosing and then dosed for 8 weeks. For E5 and E6, mice were dosed by oral gavage with ADBAC+DDAC chemical. ADBAC (Sigma Chemical, St Louis, MO) and DDAC (AK Scientific Inc., Union City, CA) were diluted in water to give the same compound ratio and alkyl chain lengths as in the disinfectant product (6.76% ADBAC (60% C-14, 25% C-12, 15% C-16) and 10.1% DDAC). Males were dosed every other day for 10 days before breeding to allow sufficient time for transit of exposed sperm into the epididymis (Dadoune and Alfonsi, 1984). Females were dosed once on the morning of GD 8, the time of neural tube closure. Doses were selected based on a preliminary dose finding study (data not shown). In E5, there was no ambient ADBAC+DDAC exposure and males were dosed at 30 mg/kg while females were dosed at 15 mg/kg. In E6, the dose was lowered to 7.5 mg/kg for both males and females to remain below the LOAEL due to the additive ambient exposure from disinfectant use in the room.

### EVALUATION OF MALFORMATIONS

For each experiment, pregnant females in both exposure and control groups were euthanized by an overdose of CO_2_ within approximately 1 hr of each other. For GD 9.5 to 10 embryos, the uterus was removed and fixed in 10% buffered formalin. After fixation, decidua were removed and the embryos dissected. All embryos and fetuses were assessed with the evaluator blind to treatment group. Each embryo was evaluated for stage of development, somite number, closure of the neural tube, the presence of pharyngeal arches, cardiac prominence and limb buds. The GD 18 fetuses were detached from the uterus; the fetal membranes were removed and the fetus and placenta weighed separately. Resorptions were characterized as early or late based on size; mummified fetuses were included as late resorptions. Fetuses were evaluated for gross morphologic lesions and fixed in 95% ethanol. Fetuses were cleared, in potassium hydroxide and stained with alcian blue and alizarin red to visualize the skeleton (Hrubec et al., 2006a). Fetuses were photographed (SZX7 sterozoom microscope with Ilumina camera, Olympus, Center Valley, NJ) and the axial skeletons evaluated for head length, vertebral, rib and sternal number, and malformations using Image-J (Hrubec et al., 2006a).

### ASSESSING CAGE CONTAMINATION

In E2, ADBAC residues in mouse boxes were analyzed using liquid chromatography with ultra-violet detection (LC/UV). DDAC cannot be measured by UV and was not determined. New unused boxes and boxes after a week’s use from each dose group were tested. Cage extracts were obtained as described previously by Melin et al. (2014) using LC/MS grade methanol (Fisher Scientific, Waltham, MA). Two composite samples were made for each dose group; each composite sample contained the extract of five boxes. Each composite sample was run in duplicate. ADBAC standards for each alkyl chain length were purchased (C12, C14, C16; Sigma Aldrich, St. Louis, MO) and dissolved in water to 1, 5, and 10 ppm. C18, which is not present in the ADBAC disinfectant, was used at 10 ppm as an internal standard to calculate recovery.

The ADBAC extraction procedure was performed as follows: 5 ml of the cage extract was evaporated and C18 internal standard, and 1 ml water was added to the residue. This solution was loaded onto an Oasis WCX solid-phase extraction column (Waters Corporation, Milford, MA) conditioned with 1 ml of methanol and 1 ml of water. The column was then washed once with 1 ml of 5% ammonium hydroxide, and twice with 1 ml of methanol. The ADBAC was eluted with 1 ml of 2% formic acid in methanol, evaporated, and reconstituted in 100 *μ*l of water. The sample was then analyzed by high performance liquid chromatography with a UV detector (Agilent Technologies Zorbax Eclipse C8 150 ×4.6, 5 *μ*, 1 ml/min flow rate) at 280 nm.

### EXPERIMENT 1: IDENTIFICATION OF POSSIBLE TERATOGENICITY

NTDs were first observed in control animals in mice and rats housed in Room A1 (*N* = 13 litters of mice and 20 litters of rats). The appearance of NTDs in GD 10 mouse embryos and GD 11 rat embryos coincided with a switch in the disinfectant used in the rodent room to one containing ADBAC+DDAC. To eliminate exposure to ADBAC+ DDAC, animals were moved to Room A2, raised for two generations, and NTDs evaluated again (*N* = 9 litters mice and 9 litters rats).

### EXPERIMENT 2: INITIAL TERATOGENICITY STUDY

To directly assess the teratogenic effect of ADBAC+DDAC, mice reared in Room A2 were randomly divided at 6 to 8 weeks of age into three groups of 8 males and 24 females per group. One group received undosed gel diet while the other groups received gel diet with 60 or 120 mg/kg/day ADBAC+DDAC for 8 weeks. Males and females were then combined for mating; mice continued to receive dosed gel diet throughout mating and gestation. For each group, half the females were evaluated on GD 10 (*N* = 10–12 per treatment) and half on GD 18 (*N* = 10–11 per treatment).

### EXPERIMENT 3: MULTIGENERATIONAL STUDY

To evaluate the persistence of ADBAC+DDAC teratogenicity, NTDs were evaluated in mice moved to a facility using chlorine dioxide disinfectant (Facility B) and compared to animals remaining in Room A2. After a 10-day acclimation period, mice in both colonies were bred to generate 15 pregnancies that were evaluated on GD 10 for the presence of NTDs. This evaluation of NTDs was repeated for F1 and F2 generation animals born in Facility B. Although the F0 generation was ambiently exposed to ADBAC+ DDAC while held in Room A2 before the move to Facility B, none of the mice in this experiment were experimentally dosed with ADBAC+DDAC.

### EXPERIMENT 4: MALE AND FEMALE CHRONIC EXPOSURE STUDY

To determine whether ADBAC+DDAC caused NTDs through male or female exposure, a study was designed to test pairings of exposed and unexposed mice. Fifteen males and 45 females were transferred from Facility B (no ambient exposure) to Room A1 (ambient exposure) and dosed with 120 mg/kg/day ADBAC+DDAC in Nutragel diet for 8 weeks. As a control, the same numbers of mice were fed undosed gel diet for 8 weeks in Facility B. After 8 weeks, 10 dosed males and 30 dosed females were transferred back to Facility B and paired for breeding to generate the following five groups: 5 undosed males bred to 15 undosed females (UMUF), 5 undosed males bred to 15 dosed females (UMDF), 5 dosed males bred to 15 undosed females (DMUF), 5 dosed males bred to 15 dosed females (DMDF), and 5 dosed males bred to 15 dosed females with dosing continuing through gestation (DMDF+G). Embryos were evaluated for NTDs on GD 10. To spread the sampling schedule of pregnant mice, groups DMDF and DMDF+G were delayed by 3 weeks from the start of the other groups. In the 3 weeks between evaluating embryos of the first three treatment groups and embryos from DMDF and DMDF+G, the animal care staff discontinued use of the ADBAC+DDAC disinfectant in Room A1 and switched to chlorine dioxide disinfectant.

### EXPERIMENT 5: MALE AND FEMALE ACUTE EXPOSURE STUDY

To clarify the contribution of paternal and maternal exposure to NTDs, we conducted an additional male and female exposure study, this time dosing by oral gavage. At 6 to 8 weeks of age, 10 male mice were dosed every other day for 10 days with 30 mg/kg/day ADBAC+DDAC. After dosing, the 10 males were bred with 30 unexposed females. On GD 8, half of the pregnant females bred to the dosed males were given a single oral gavage dose of 15 mg/kg (DMDF), while the other 15 received saline (DMUF). Simultaneously, 10 males received saline gavage every other day for 10 days and were then bred to 30 females. Fifteen pregnant females were transferred to Room A3 on GD 3 and were dosed with 15 mg/kg ADBAC+DDAC on GD 8 (UMDF). Fifteen pregnant females were dosed with saline on GD 8 (UMUF). All dams were killed at GD 9.5 and embryos collected and evaluated for NTDs as described above.

### EXPERIMENT 6: DOSED AND AMBIENT EXPOSURE STUDY

To clarify the contribution of ambient exposure on NTDs in ADBAC+DDAC exposed mice, we compared NTDs in offspring of dosed mice with that of mice receiving only ambient exposure to the disinfectant in the mouse room. Ten males and 30 females were transferred from Facility B to Facility C where they received ambient exposure from ADBAC+DDAC disinfectant use in the mouse room for 10 days. In addition to the ambient exposure, 5 males were dosed every other day for 10 days by oral gavage with 7.5 mg/kg ADBAC+DDAC. The mice were bred and the dosed group females received a single gavage of 7.5 mg/kg on GD 8. Five males and 15 females remained in Facility B as controls and were gavaged with saline on the same schedule as the dosed group. All dams were killed at GD 9.5 and embryos collected and evaluated for NTDs as described above.

### STATISTICAL ANALYSIS

All experiments were conducted with the mother as the experimental unit; thus the *N* for each experiment is the number of litters in a treatment group. All determinations were averaged first per litter and then all litter values were averaged by treatment. The NTD data and morphometric data from GD 18 mouse fetuses were analyzed by analysis of variance (ANOVA) (Statistix, Tallahassee, FL) followed by a Dunnett’s Multiple Comparison Test with significance set at *p* < 0.05. Variance in fetal placental ratio was evaluated by an F-Test based on litter averages in each treatment with alpha set at 0.05.

## Results

Initial findings at WSU suggested that QAC disinfectants increased late fetal demise and altered pup numbers; additionally, a large range in developmental stage was observed in late gestational fetuses. These findings prompted the following six experiments (numbered E1 to E6) at VA Tech. The first experiment (E1) identified QAC disinfectants as the likely cause of NTDs in both mice and rats. E2 demonstrated a dose response relationship between disinfectant exposure and the formation of NTDs. E3 revealed the persistence of a multigenerational effect from QAC exposure. E4 and E5 demonstrated the contribution of ambient exposure on NTD formation and also showed that either maternal of paternal exposure was sufficient to induce NTDs in offspring. E6 demonstrated that ambient use of the disinfectant in the mouse room was a greater driver of NTD formation than dosing.

**FIGURE 2:**
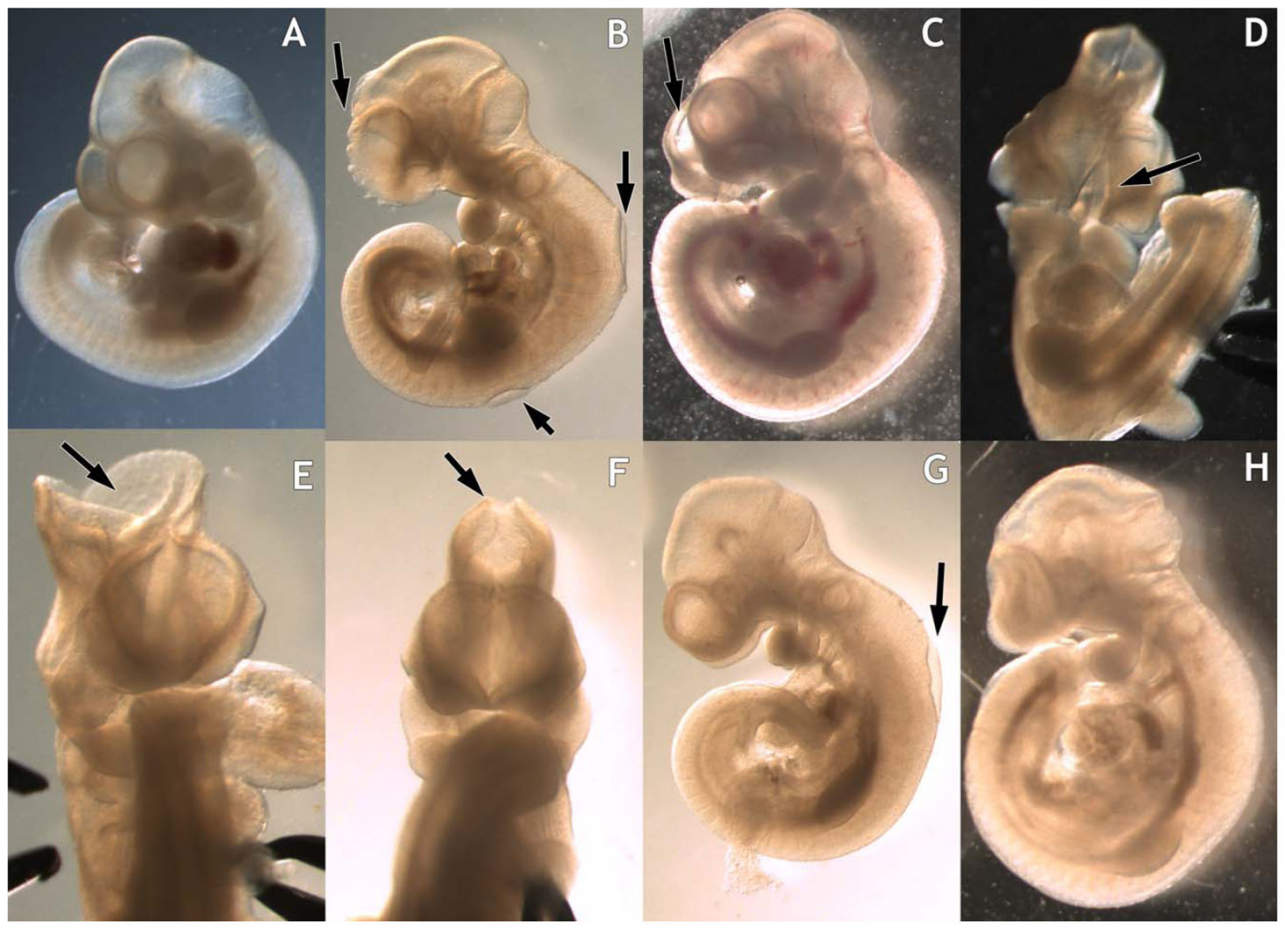
Neural tube defects (NTDs) in GD10 mouse embryos exposed to ADBAC+DDAC. **A**: Control unexposed. **B–D**: Incomplete closure of the rostral face, B also has two spinal NTDs. **E,F**: Cranial NTDs demonstrating a range in defect size. **G**: Spinal NTD. **H**: Embryo with altered morphology in the absence of open NT.

The appearance of NTDs originally coincided with the switch to a disinfectant cleaner containing ADBAC+DDAC. NTDs were located at the three closure sites described in mice as shown in Figure 2 (Copp and Greene, 2013). Incomplete closure of the rostral face at the rostral closure site resulted in a split face lesion. In some individuals, the entire face was split. Cranial lesions were observed at the midbrain closure site and ranged in size from small to large openings. Spinal lesions were observed mainly at the hindbrain spinal boundary, but were also observed at other locations along the spine.

Some embryos had multiple defects with incomplete closure at two or more locations and some also exhibited an abnormal phenotype, appearing compressed and misshapen in the presence of an intact NT (Fig. 2).

**FIGURE 3:**
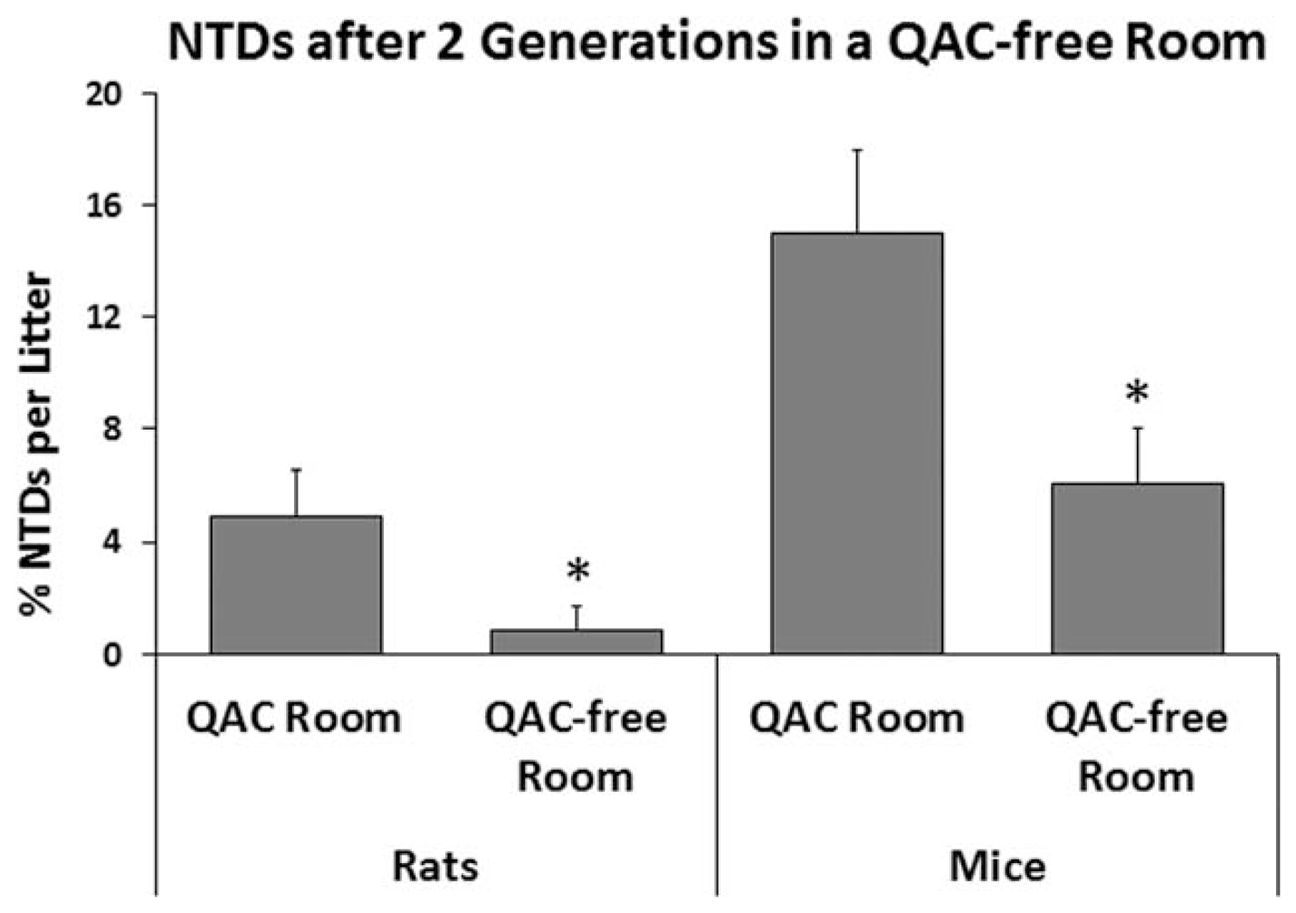
Experiment 1: NTDs in GD 11 rat and GD 10 mouse embryos exposed to ambient ADBAC+DDAC disinfectant from normal husbandry practices and after being raised in a room using ethanol and chlorine dioxide. NTDs were reduced after being removed from the ADBAC+DDAC environment (* indicates significant difference between the two rooms, ANOVA *p* ≤ 0.05). Values represent the mean ± SEM percent per litter with *N* = 20 rats and 13 mice in the QAC room, and *N* = 9 rats and 9 mice in the QAC-free room.

**FIGURE 4:**
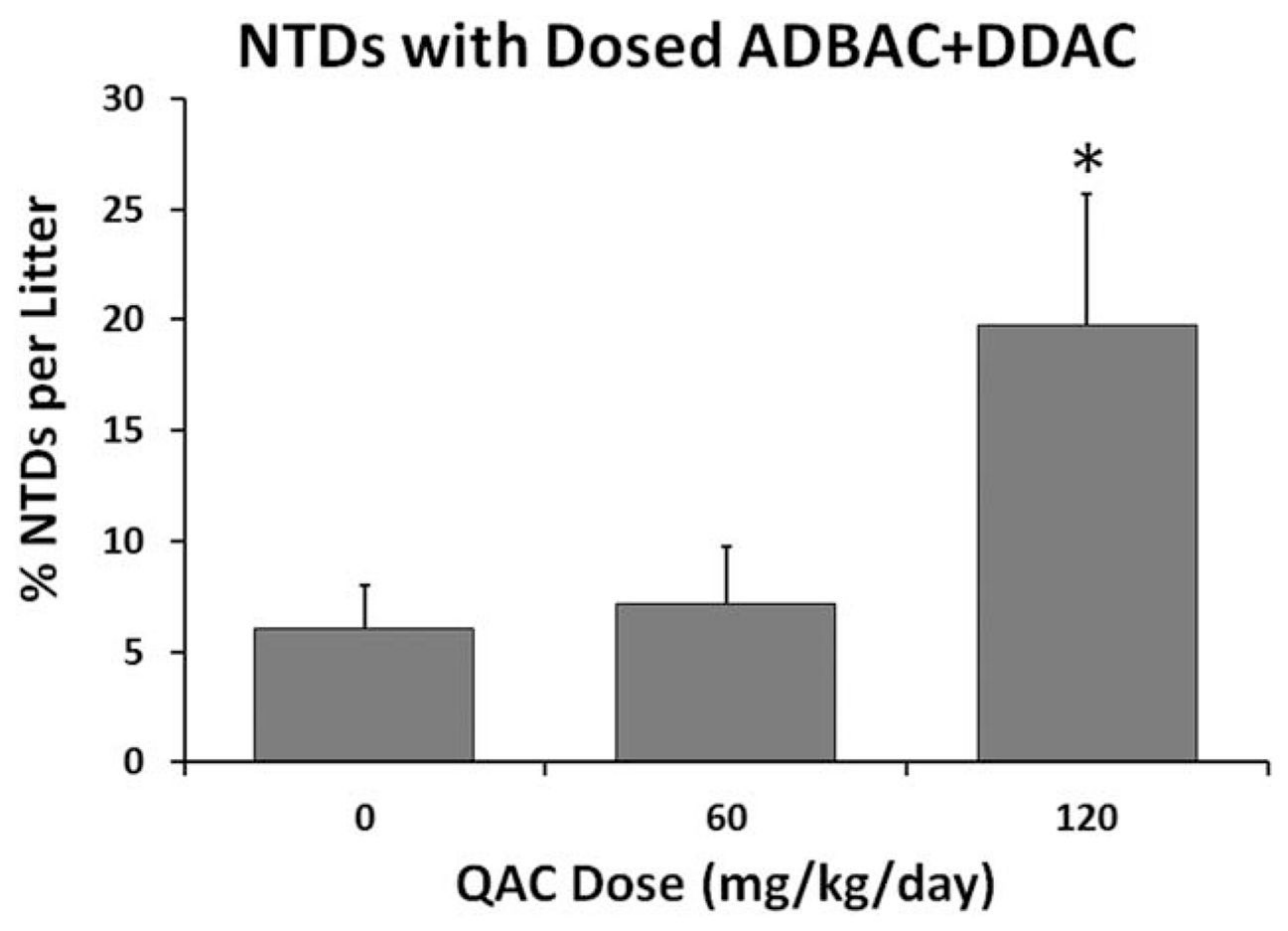
Experiment 2: NTDs in GD 10 mouse embryos dosed in the feed with ADBAC+DDAC disinfectant for 8 weeks. The numbers of NTDs were significantly greater in the 120 mg/kg/day exposed group compared with controls (* indicates significant difference from control, ANOVA *p* ≤ 0.05). Values represent the mean ± SEM percent per litter with *N* = 10 to 12 dams per treatment.

After identifying the disinfectant as the possible teratogen, we created a “QAC free” room (A2) and raised rats and mice for two generations and evaluated NTDs again (E1). Both species exhibited significant declines in NTDs after the move to Room A2 (Fig. 3). The prevalence was higher in mice than in rats both before and after moving to the “QAC free” environment, indicating a possible difference in species sensitivity. Concurrently, in E2 in which embryos were evaluated from mothers dosed with 60 or 120 mg/kg/day disinfectant, we found significantly higher levels of NTDs among the offspring of mothers dosed with 120 mg/kg/day (Fig. 4). However, in both E1 and E2, control mice displayed NTDs, suggesting continued environmental exposure. Thus, although the correlation between dose and the incidence of NTDs strongly suggested that QACs were the cause of NTDs, the presence of NTDs in controls prevented proof of concept.

**FIGURE 5:**
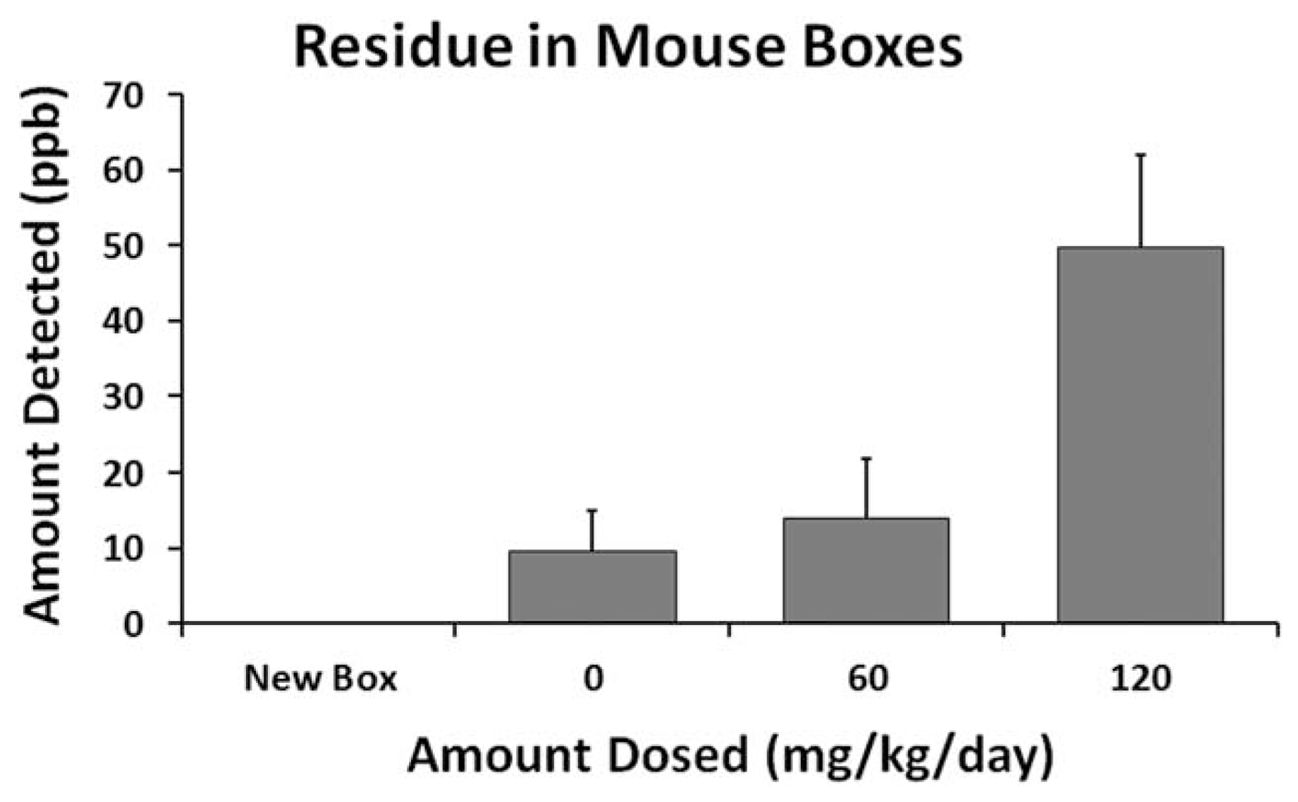
ADBAC residues detected in mouse boxes. ADBAC residues from new or used mouse boxes were extracted with methanol and analyzed by liquid chromatography with ultra violet detection. New boxes did not contain ADBAC residues. Residues were detected in all used boxes. Residues in the boxes of control mice (0 mg/kg/day) indicated inadvertent exposure in the room. *N* = 2 samples for each treatment. Each sample contained the residue from five boxes. Each sample was run in duplicate and averaged.

To determine if the control mice in Room A2 were being exposed inadvertently, we measured ADBAC residues in the boxes of control and dosed mice. ADBAC could not be detected in new unused boxes; however, residues were present in all boxes that housed mice (Fig. 5). As expected, mice dosed at 120 mg/kg/day had the highest residues of ADBAC present in their boxes. Residues in boxes of control mice from Room A2 were similar to those in boxes housing mice dosed at 60 mg/kg/day in Room A1. Control mice in Room A2 were being inadvertently exposed despite our best efforts to prevent it.

A similar experience occurred in Dr. Hunt’s laboratory at WSU. In that instance, an entire “QAC-free” rodent facility became contaminated when a QAC dosing study was initiated. The cage wash system was identified as the source of contamination, spreading ADBAC+DDAC residues from experimental caging to clean boxes, and thus exposing mice throughout the facility. Importantly, despite the fact that the WSU facility did not use QAC disinfectants, ADBAC+DDAC exposure from contaminated caging was sufficient to reduce litter size. Litter sizes in nonexperimental mice decreased from an average of 9.2 pups per litter to 8.2. The WSU study was immediately terminated and litter sizes rebounded to 8.9 pups per litter the following month.

The contamination events in both the WSU and VA Tech studies highlight the necessity to isolate dosed and undosed mice. Housing control and exposed mice in separate rooms within the same facility resulted in exposure of controls at both institutions and was not sufficient. Once control mice were removed from the facility that used QAC disinfectants and the exposure was eliminated, the negative control was re-established and NTDs were no longer seen in control mice.

**FIGURE 6:**
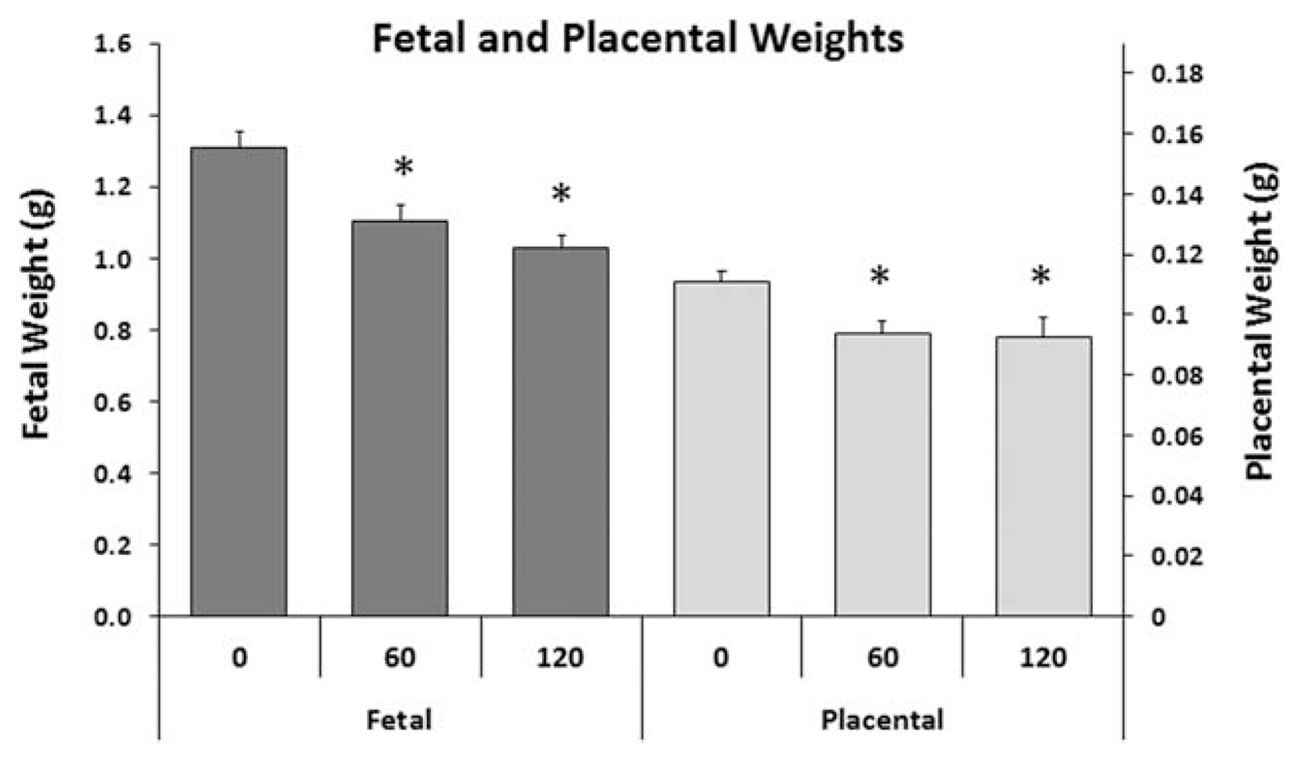
Experiment 2: Average fetal weight and placental weight of GD 18 fetuses in mice dosed in the feed with ADBAC+DDAC disinfectant for 8 weeks. Fetal and placental weights were significantly lower in 60 and 120 mg/kg/day exposed mice compared with controls (* indicates significant difference from control, ANOVA *p* ≤ 0.05). Values represent the mean ± SEM with *N* = 10 to 11 dams per treatment.

**FIGURE 7:**
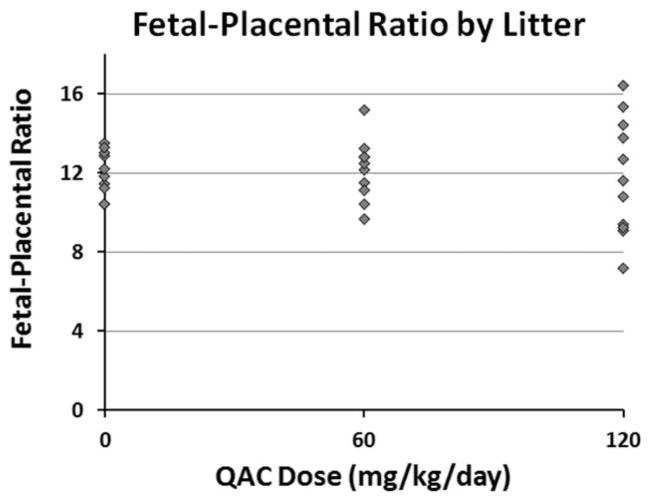
Experiment 2: Distribution of average fetal–placenta ratio for GD 18 litters dosed in the feed with ADBAC+DDAC disinfectant for 8 weeks. While the mean ratios were quite similar in each treatment group, the range was significantly greater in the 60 and 120 mg/kg/day exposed litters (F-test, *N* = 10–11 dams per treatment).

In addition to measuring NTDs in E2, we also evaluated late gestation fetuses on GD 18 from the same cohort of mice. Both fetal weight and placental weights were significantly reduced in 60 and 120 mg/kg/day ADBAC+DDAC exposure groups compared to controls (Fig. 6); however, due to the inadvertent exposure of control litters described above, the effects of ADBAC+DDAC on fetal and placental weights may actually be even greater. Reduced fetal growth can be the result of placental insufficiency, therefore, the fetal–placental ratio was evaluated (Fig. 7). There was no difference in the mean fetal placental ratio between control and treated mice; however, there was a significant increase in the variance of the ratio at 60 and 120 mg/kg/day as demonstrated by the wider distribution of ratios in dosed litters.

No gross malformations were observed in the GD 18 fetuses. Evaluation of the axial skeleton in clear stained fetuses revealed few malformations (Table 2). Only lumbar rib ossification centers on the right were significantly increased with ADBAC+DDAC exposure. While lumbar rib ossification centers without cartilage can increase with environmental exposures, they are not considered malformations, but rather transient structures that likely become incorporated into the lumbar transverse process (Chernoff and Rogers, 2004). Fetal demise (total resorptions) was increased to 17.1% at 120 mg/kg/day (Table 2). This was not significantly different from controls, possibly due to inadvertent exposure of control litters. Fetal demise in late gestation (late resorptions) at 120 mg/kg/day was significantly elevated at 12.4% compared with 3% in control litters. This increase in fetal death could be the reason why no gross malformations were observed in the GD 18 fetuses.

**FIGURE 8:**
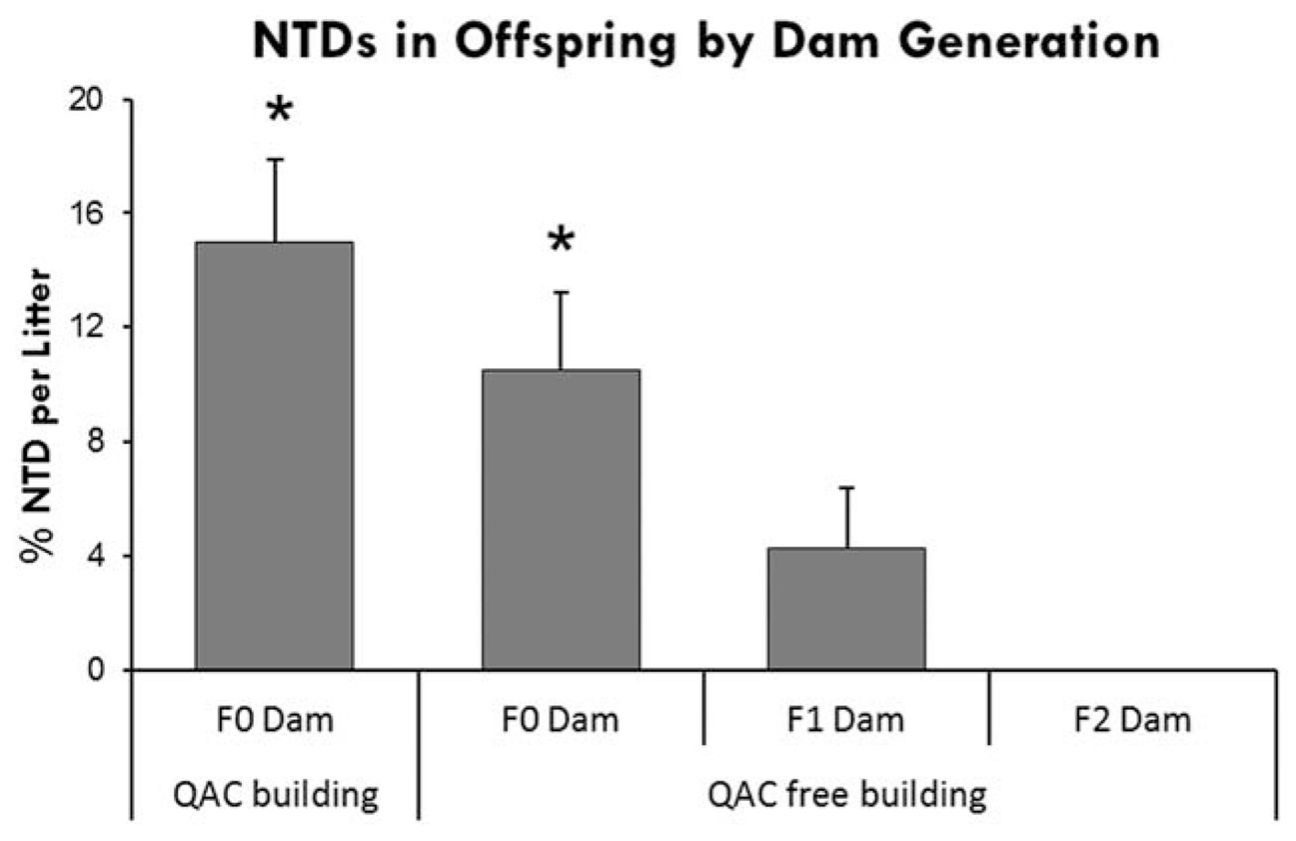
Experiment 3: NTD malformations in GD 10 embryos followed for three generations after moving to a facility that did not use QAC containing disinfectants. F0 mice were exposed to ambient disinfectant in the QAC facility. F0 females moved to a QAC-free facility, had fewer NTDs than the same cohort remaining in the QAC facility. Subsequent generations in the QAC-free facility exhibited a decline in NTDs (* indicates significant difference from F2 Dams, ANOVA *p* ≤ 0.05). Values represent the mean ± SEM percent per litter, *N* = 12 to 15 dams per generation.

To test the persistence of NTDs after the cessation of exposure, in E3 we monitored NTDs in offspring for three generations after animals were moved to a QAC-free facility (Facility B). As in E1, NTDs declined in the offspring of F0 dams by comparison to the same cohort remaining in Room A2 (Fig. 8). A further decline was evident in the offspring of F1 dams, and NTDs were not observed in the offspring of F2 dams. Because the F0 generation received ambient exposure to disinfectant before the move to Facility B, this experiment demonstrates that ambient exposure alone is sufficient to cause NTDs that persist for multiple generations after cessation of exposure.

**FIGURE 9:**
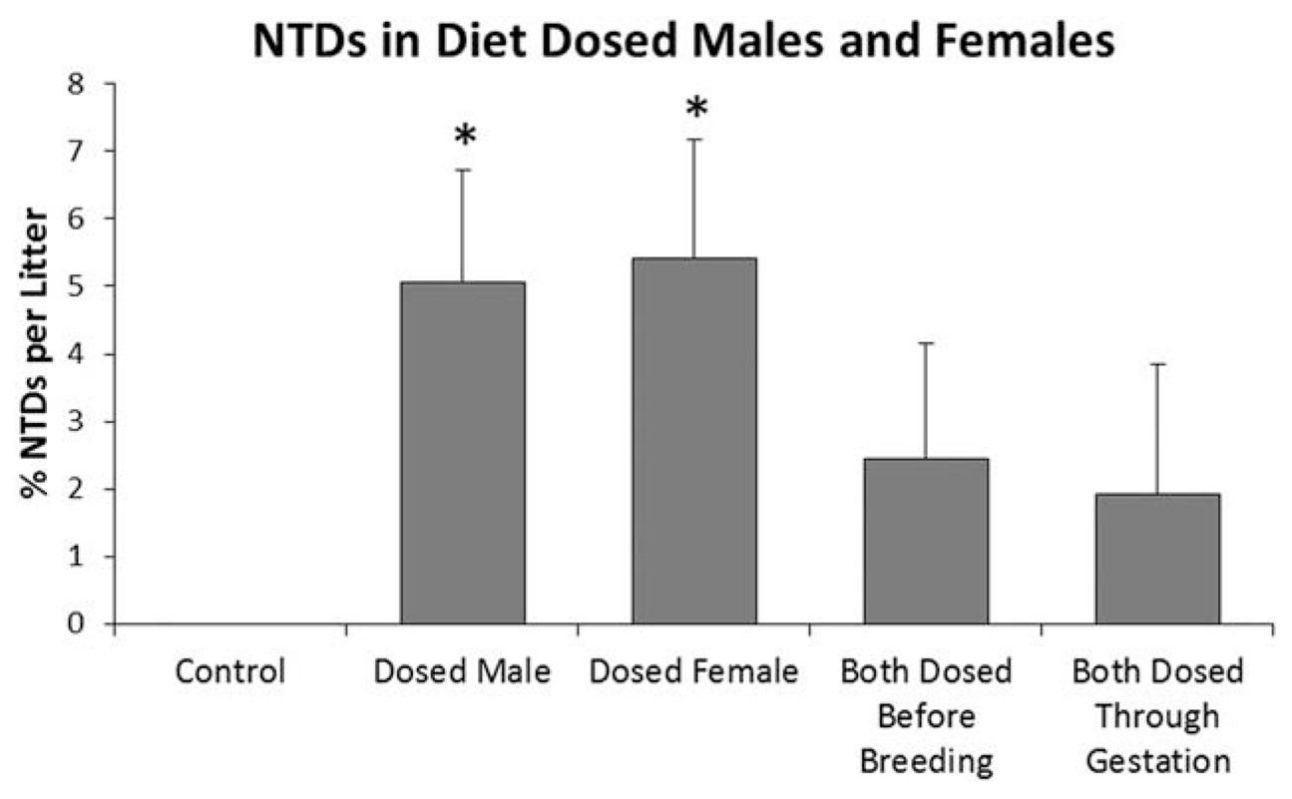
Experiment 4: The role of maternal and paternal ADBAC+DDAC exposure on NTDs in GD 10 in offspring. One or both parents were dosed with 120 mg/kg ADBAC+DDAC in the feed for 8 weeks. Mice were moved to a non-QAC facility for breeding and gestation except one group that continued exposure of both parents throughout breeding and gestation. NTDs were significantly higher when only a single parent was dosed; however, husbandry use of ADBAC+DDAC disinfectant was discontinued in between evaluation of single parent and both parent groups. Removal of the ambient exposure likely resulted in lower NTDs in the groups with both parents dosed. (* indicates significant difference from controls, ANOVA *p* ≤ 0.05). Values represent the mean ± SEM percent per litter, *N* = 12 to 15 dams per treatment group.

The role of maternal and paternal ADBAC+DDAC exposure in the genesis of NTDs was evaluated in E4. NTDs were observed in offspring when only males or only females were dosed, as well as when both parents were dosed (Fig. 9). Unexpectedly, levels of NTDs were lower when both parents were dosed than when a single parent was dosed and regardless of whether exposure ended before mating or continued throughout mating and gestation. This finding was puzzling until we realized that the animal care staff had discontinued use of the ADBAC+ DDAC disinfectant in Room A1 toward the end of our study. For logistical reasons, groups with separately dosed males and females were offset by 3 weeks from the groups in which both parents were dosed. ADBAC+DDAC use in the mouse room was discontinued in the 1-week interval after embryos from the single parent dose groups were evaluated, but before the groups with both parents dosed were paired for breeding. Although the change in environmental exposure prevents direct comparison between groups, the results of this study provide clear evidence that either maternal or paternal exposure is sufficient to induce NTDs. The results also provide further support that ambient exposure significantly influences the rate of NTDs.

**FIGURE 10:**
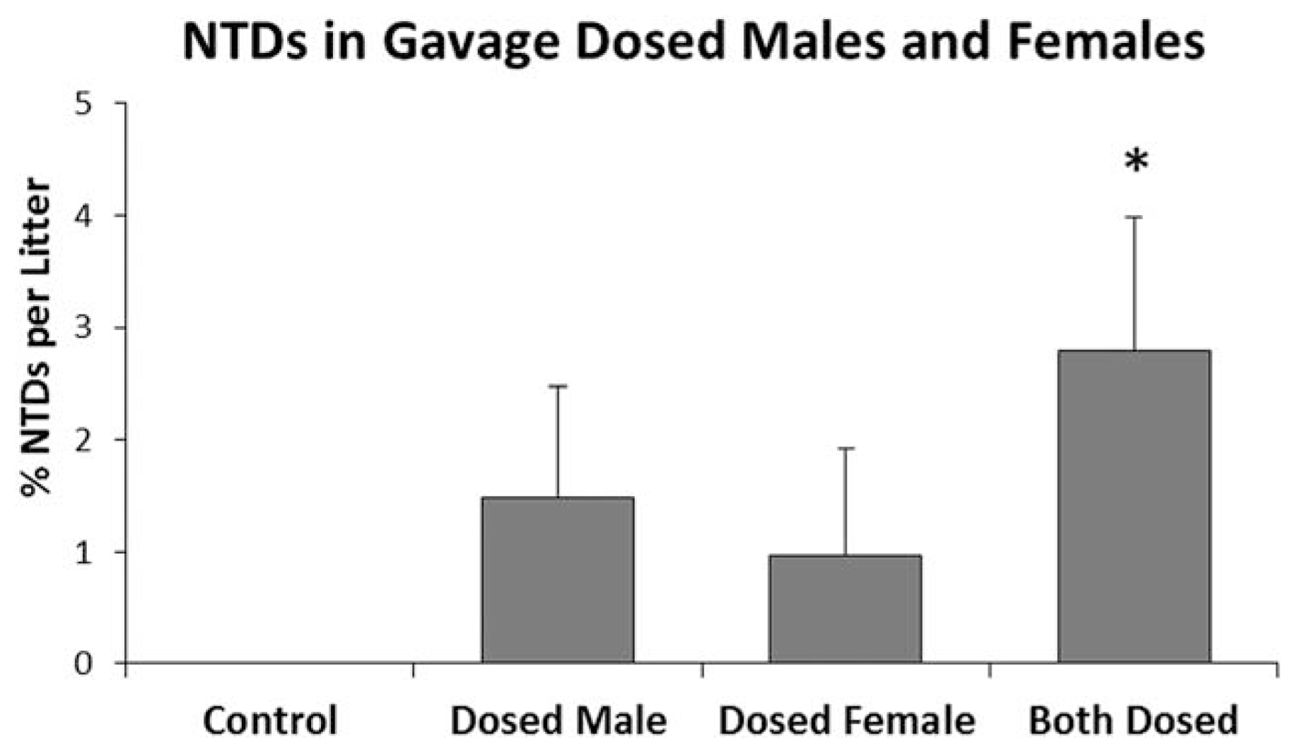
Experiment 5: The role of maternal and paternal ADBAC+DDAC exposure on NTDs with no ambient exposure from disinfectant use in the mouse room. Males were dosed every other day for 10 days with 30 mg/kg, and females were dosed once on GD 8 with 15 mg/kg ADBAC+DDAC. NTDs were significantly higher when both parents were dosed (* indicates significant difference from controls, ANOVA *p* ≤ 0.05). Values represent the mean ± SEM percent per litter, *N* = 12 to 15 dams per treatment group.

When maternal and paternal only exposure was reevaluated under consistent environmental conditions (E5), NTDs were observed in all dosed groups and were highest when both parents were exposed (Fig. 10), suggesting that the effects of exposure are additive. In this experiment, however, levels of NTDs did not reach significance when only a single parent was dosed and, when both parents were dosed, the incidence of NTDs was far lower than the 15% per litter observed in E1. The reduction in NTDs could be due to the short-term dosing; males received five doses prebreeding, while females received one dose on GD 8. In previous studies, mice were dosed at higher concentrations for 8 weeks in the diet. NTDs could also be lower in this experiment because mice were no longer receiving ambient exposure from ADBAC+DDAC disinfectant use in Room A3.

**FIGURE 11:**
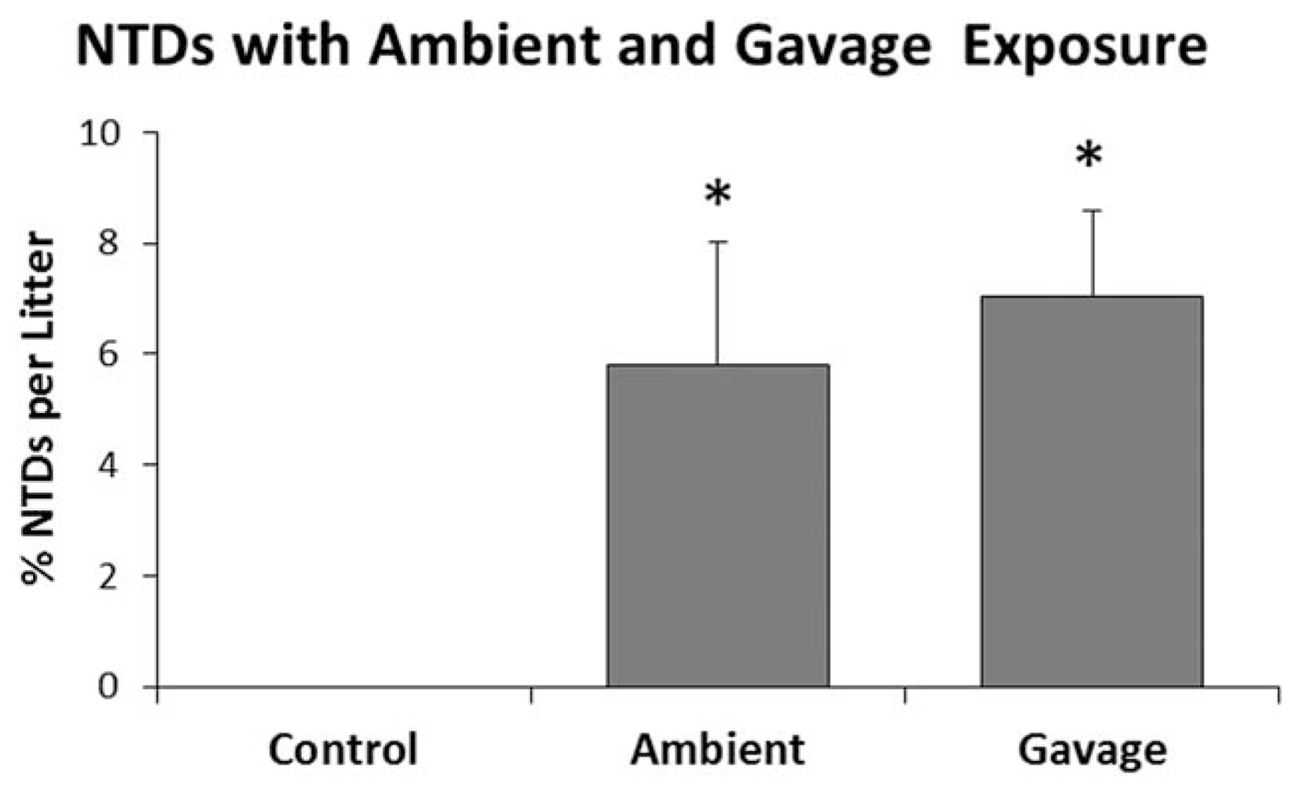
Experiment 6: NTDs in mice exposed to ambient husbandry use of the disinfectant or ambient plus 7.5 mg/kg ADBAC+DDAC by gavage. Males were dosed every other day for 10 days, and females were dosed once on GD 8. NTDs were significantly higher than controls. There was no difference between mice receiving ambient or ambient+gavage exposure (* indicates significant difference from controls, ANOVA *p* ≤ 0.05). Values represent the mean ± SEM percent per litter, *N* = 12 to 15 dams per treatment group.

To confirm that ambient exposure as a result of disinfectant use in the mouse room contributed significantly to NTD formation, we moved mice for E6 to Facility C, a facility using ADBAC+DDAC disinfectants. Mice received ambient exposure for 2 weeks or ambient+gavage. Both ambient and ambient+gavage groups exhibited significantly increased levels of NTDs compared with unexposed controls (Fig. 11); however, the difference between mice receiving ambient exposure and those receiving ambient+gavage was not significant. Importantly, despite the fact that mice in E6 received a lower dose, the incidence of NTDs was more than twice that seen in E5. These date clearly demonstrate the important contribution of ambient exposure in NTD formation.

## Discussion

Previously, we demonstrated that ADBAC+DDAC exposure causes reproductive toxicity in both male and female mice (Melin et al., 2014, 2016). The present study describes developmental toxicity in both mice and rats that manifests as NTDs in early gestation and decreased pup size and survivability in late gestation. NTDs were seen with ADBAC+DDAC dosed acutely by oral gavage, chronically in feed, and ambiently through the use of disinfectant in the mouse room. Maternal and paternal toxicity was not observed at the doses used in these experiments. A dose effect was observed with both oral and ambient exposures; increased exposure resulted in higher levels of NTDs. While ambient exposure could not be quantified, the use of ADBAC+DDAC disinfectant increased NTDs and contributed to exposure.

Every year in the United States, 150,000 to 200,000 babies (3.3–5.6% of live births) are born with a structural birth defect. This figure increases to 17% to 20% of live births when neurobehavioral and learning disabilities are included (Boyle et al., 2011). NTDs are the second most prevalent malformation after cardiac defects (Christianson et al., 2006). In our studies, NTDs were seen in both rats and mice following ambient exposure to the QAC-containing disinfectant in the mouse room. NTDs were also observed in mice dosed with the disinfectant at 60 or 120 mg/kg/day in feed, or with ADBAC+DDAC chemical by gavage at 7.5, 15, or 30 mg/kg/day. Thus, our data suggest that malformations are induced at relatively low, environmentally relevant doses.

ADBAC and DDAC are used extensively in household, medical, and industrial settings, as well as in restaurants and food production facilities. Over 1 million pounds each of ADBAC and DDAC are manufactured in the United States each year (PubChem Database). QAC disinfectants are generally viewed as having low toxicity; and although human deaths have been reported from accidental overdose, these events are rare (Chataigner et al., 1991; Ellenhorn et al., 1997). Chronic exposure is known to cause asthma and dermatitis (Bernstein et al., 1994; Suneja and Belsito, 2008), as well as ocular inflammation and hypersensitivity (Hong and Bielory, 2009). The separate ADBAC and DDAC components have been tested for regulatory purposes in reproductive and developmental toxicity studies in rats and rabbits, and were found to decrease fetal weight at higher doses (Cosmetic Ingredient Review, 1989; Tyl, 1989; Neeper-Bradley, 1990, 1993). These studies also reported small litter size with an increase in resorptions and late gestation fetal death (Cosmetic Ingredient Review, 1989; Tyl, 1989; Neeper-Bradley, 1993). Malformations were not observed with the exception of one study that found increased sternal abnormalities in benzalkonium chloride exposed rat fetuses (Cosmetic Ingredient Review, 1989). Our study is the first to evaluate the developmental toxicity of ADBAC+DDAC combination.

Before introduction of ADBAC+DDAC disinfectants in the mouse room, the background rate for NTD malformations in our CD-1 embryos was approximately 1/1000 (Mallela et al., 2011). Following introduction of QAC disinfectants, ambient exposure resulted in 15% of embryos and 100% of litters affected at GD 10. Early gestation embryos were not evaluated in the regulatory studies discussed above; however, our results for GD 18 fetuses showing decreased fetal weight, increased resorptions, increased late gestational death, and few if any fetal malformations, matched the findings of the regulatory studies for the individual ADBAC and DDAC components (Cosmetic Ingredient Review, 1989; Tyl, 1989; Neeper-Bradley, 1990, 1993).

Despite the NTDs observed in GD 10 embryos, we did not see exencephaly in late gestation fetuses. This was unexpected as NTDs are not lethal to the developing fetus. It is possible that increased fetal death from ADBAC+ DDAC exposure could explain the lack of malformations in GD 18 fetuses. The regulatory studies and previous studies from our laboratory found increased resorptions and late gestation fetal death (Cosmetic Ingredient Review, 1989; Tyl, 1989; Neeper-Bradley, 1993; Melin et al., 2014, 2016). In the present study, total resorptions were increased to 17.1% in 120 mg/kg/day litters which is close to the number of NTDs observed at GD 10. Total resorptions were not statistically significant; however, late resorptions were significantly higher at 120 mg/kg/day than controls. The lack of significance with total resorptions could be due to inadvertent exposure of controls. Total resorptions in control litters were 9.4% which is higher than the lab’s historic rate of 5% (Hrubec et al., 2006b). A few treated litters also exhibited 100% mortality as described previously in Melin et al. (2014, 2016); these litters were not included in the analysis presented in this study as gestational stage could not always be determined. Additionally, some exposed embryos exhibited an abnormal phenotype, appearing compressed and misshapen. This could indicate further toxicity that could be contributing to fetal demise of affected fetuses. In aggregate, these studies clearly demonstrate increased fetal death, particularly in late gestation, following ADBAC+DDAC exposure and could be a reason for the lack of exencephaly in GD 18 fetuses.

An alternative explanation for lack of malformations in late gestation fetuses is that the NTDs observed at GD 10 represent developmental delay. If so, then the open NT would close if allowed to develop further. Both the Hrubec and Hunt labs observed an increase in the range of developmental stages present within a litter and also an increase in the number of runts born following ADBAC+D-DAC exposure. This suggests dysregulation of temporal events during development and the possibility for developmental delay. While only age appropriate developed embryos were analyzed, NT closure delay can occur in otherwise normally developed embryos and some of the NTDs could represent developmental delay. Delayed neural tube closure is a cause for concern by itself; not only can delay result in a NTD, it also indicates that normal developmental sequences in the central nervous system are disrupted.

Altered timing of critical developmental events could manifest as functional or behavioral defects in the adult. Compounds associated with increased risk for NTDs, such as valproic acid, carbamazepine, and polycyclic aromatic hydrocarbons, also cause neurobehavioral disorders and cognitive delay (Meador et al., 2007; Perera et al., 2012; Wang et al., 2015). It is also possible that some form of repair process is occurring during gestation that reduces or eliminates the NTDs in GD 18 fetuses. Further studies are needed to determine if some or all of the NT lesions observed at GD 10 close over time, or whether affected fetuses die later in gestation.

Decreased fetal weight was observed in this study which could indicate direct developmental toxicity. Alternatively, decreased fetal growth can occur from a mismatch between fetal and placental growth. If the placenta is too small relative to the fetus, the fetus receives insufficient nutrition. Conversely, an overlarge placenta can siphon nutrients away from the developing fetus and result in reduced fetal growth. We observed significant variation in the fetal placental ratio at both ends of the mismatch spectrum at both the 60 and 120 mg/kg/day doses. This mismatch could also be contributing to the increase in ADBAC+DDAC associated late gestation fetal demise observed at both WSU and at VT (Melin et al., 2014).

In human epidemiological studies, both small and large placentas relative to birth weight have been associated with fetal death and preterm deliveries (Haavaldsen et al., 2013). Abnormal placenta-to-birth-weight ratio has been associated with adult disease; specifically, disproportionately large placentas relative to birth weight have been associated with increased risk of death from cardiovascular disease (Risnes et al., 2009). Placental development is orchestrated primarily through imprinted genes. The conflict hypothesis proposes that paternal genes enhance, and maternal genes suppress, fetal growth. Imbalances in expression of imprinted genes in the placenta are associated with restricted fetal or placental growth in both humans and mice (Coan et al., 2005). The large variation in fetal placenta ratio seen in ADBAC+DDAC treated litters could be due to differences in epigenetic imprinting of placental genes; although further studies are needed to test this possibility.

Alternatively, endocrine disrupting compounds, such as bisphenol A and phthalates, alter mouse placental architecture, size and function (Vrooman et al., 2016). Previously we demonstrated that QACs alter normal reproductive capabilities and estrus cycling in mice, thus acting as endocrine disruptors (Melin et al., 2014, 2016). Changes in placenta size and growth could be a result of altered hormonal or endocrine signaling in the maternal/placental/fetal unit. Given the importance of the placenta to both maternal and fetal health, the effects of QAC disinfectants on the placenta should be studied further.

Perhaps the most important finding from this study was the presence of NTDs following male only exposure. Certain paternal occupations have been associated with an increased risk for birth defects in offspring; however, epidemiological studies often could not identify the specific exposure responsible (Trasler and Doerksen, 1999; Shaw et al., 2002). More recently, epidemiological studies have linked paternal exposures to classes of chemicals such as polychlorinated compounds, pesticides, and organics such as benzene, turpentine, diesel fuel, and creosote with an increase in structural birth defects (reviewed by Braun et al., 2017). Animal studies have found that paternal exposure to alcohol, lead, or cyclophosphamide can result in structural or behavioral abnormalities in the offspring (reviewed by Curley et al., 2011; Anderson et al., 2014).

Male exposures can affect the offspring through genetic or epigenetic mechanisms (Anway et al., 2005; Braun et al., 2017). QACs are not mutagenic (EPA, 2006); thus, it is likely they act through an epigenetic mechanism. Epigenetic changes in the male germ line that are subsequently passed on to the offspring provide a mechanism for male-mediated teratogenesis. In this study, ADBAC+DDAC exposure did not have to occur during breeding or gestation. Male or female exposure that ended 7 to 10 days before breeding was sufficient to cause NTDs. These results emphasize the importance of considering both maternal and paternal exposures when evaluating developmental outcomes.

Equally important was the finding that ambient exposure from use of disinfectant in the mouse room had a significant impact on NTD formation. Typically, toxicity assessments first evaluate high doses to demonstrate an effect; after toxicity is demonstrated, the dose is reduced to concentrations that are clinically or environmentally relevant. This progression is often criticized because toxicity is frequently first observed at unrealistically high concentrations. The data presented here demonstrate ADBAC+ DDAC caused NTDs at unrealistically high concentrations, but also at ambient concentrations, from normal use of the disinfectant in the mouse room.

The highest levels of NTDs were seen originally in mice that had been bred and raised for multiple generations with ambient exposures (E1). When ADBAC+DDAC disinfectant use was terminated in the middle of one experiment (E4), the NTDs dropped significantly, despite the fact that mice were being provided a high dose of 120 mg/kg/day. In the last two experiments, mice receiving a lower oral dose along with ambient exposure had more NTDs than mice receiving a higher oral dose without ambient exposure. Ambient use of the disinfectant likely results in a combination of inhalation and oral exposure. The fact that ambient exposure impacts NTDs to a greater extent than oral dosing alone is not surprising. The Environmental Protection Agency’s (EPA’s) risk assessment for ADBAC and DDAC determined that inhalation exposure carries a greater risk to human health than oral routes of exposure (EPA, 2006). It is, however, sobering that ambient use of the disinfectant in the mouse room at the prescribed rate of use caused NTDs in both rats and mice.

Collectively these results beg the serious question: Do ADBAC and DDAC impact human development? Rodents are used world-wide as surrogates to evaluate potential human toxicity. The fact that we see developmental toxicity in both rats and mice from exposure through normal husbandry practices indicates a potential for human toxicity. QAC use has increased significantly over time, and products containing untested ADBAC+DDAC mixtures are now commonplace (PubChem database). These changes have modified the quantity and quality of human exposure. The magnitude of production and variety of QAC containing products ensures that people are regularly and repeatedly exposed. Increased human exposure and the scarcity of human data, coupled with observed rodent toxicity, all advocate for immediate study of the effects of QACs exposure on human health and development.

## Figures and Tables

**TABLE 1:** Facilities and Disinfectants Used in Each Experiment

Facility	Disinfectant	Species	Experiment #
A Room A1	ADBAC+DDAC	Rats, mice	Exposed rats and mice for E1 Exposed mice for E2, E4, and E5
A Room A2	Ethanol and chlorine Dioxide	Rats, mice	Control rats and mice for E1 & E2
A Room A3	Chlorine dioxide - This is Room A1 after discontinued use of ADBAC+DDAC	Mice	Exposed mice for E4 and E5
B	Chlorine dioxide	Mice	Experiment E3 Control mice for E4, E5, E6, Control breeding colony
C	ADBAC+DDAC	Mice	Exposed (dosed and ambient) for E6

**TABLE 2:** Fetal Resorptions and Axial Skeletal Analysis of Gestational Day 18 Mouse Fetuses Exposed to ADBAC+DDAC Disinfectant for 8 Weeks and Throughout Breeding and Gestation

Measurement	ADBAC+DDAC concentration					
	0		60		120	
Total resorptions	9.4	(2.7)	8.5	(2.0)	17.1	(2.7)
Early resorptions	6.4	(2.3)	2.3	(1.1)	4.7	(2.9)
Late resorptions	3.0	(2.2)	6.2	(2.1)	12.4*	**(2.3)**
# Cervical vertebrae	7	(0)	7	(0)	7	(0)
# Thoracic vertebrae	13	(0)	13	(0)	13	(0)
# Lumbar vertebrae	6	(0)	6	(0)	6	(0)
# Sacral vertebrae	4	(0)	4	(0)	4	(0)
# Sternal ossification centers	6	(0)	5.9	(0.1)	6	(0)
% Tuberculi anterior on C7 - R	0	(0)	0	(0)	0	(0)
% Tuberculi anterior on C7 - L	0	(0)	0	(0)	8.3	(8.3)
Total ribs counted	26.2	(0.1)	26.3	(0.2)	26.7	(0.2)
% Cervical ribs - R	5.0	(5)	8.3	(8.3)	8.3	(8.3)
% Cervical ribs - L	10.0	(6.7)	0.0	(0)	16.7	(10.5)
% Total cervical ribs	15.0	(10.7)	8.3	(8.3)	25.0	(11.2)
% Lumbar rib ossification center–R	0.0	(0)	8.3	(8.3)	25.0*	**(11.2)**
% Lumbar rib ossification center– L	5.0	(5)	16.7	(10.5)	16.7	(10.5)

Resorptions were characterized as early or late based on size; mummified fetuses were included as late resorptions. Fetuses were cleared in potassium hydroxide and stained with alcian blue and alizarin red. Values represent the mean (SEM). For resorptions, *N* = 10–11; for skeletal analysis, *N* = 10, 6, and 6 for 0, 60, and 120 mg/kg/day, respectively. Late resorptions and lumbar rib ossification centers on the right (R) at 120 mg/kg/day were significantly different than controls (ANOVA *p* ≤ 0.05).
